# Are Corticosteroids Beneficial for Sepsis and Septic Shock? Based on Pooling Analysis of 16 Studies

**DOI:** 10.3389/fphar.2019.00714

**Published:** 2019-07-12

**Authors:** Yang-Yang Yao, Lu-Lu Lin, Hui-Yun Gu, Jun-Yi Wu, Yu-Ming Niu, Chao Zhang

**Affiliations:** ^1^Center for Evidence-Based Medicine and Clinical Research, Taihe Hospital, Hubei University of Medicine, Shiyan, China; ^2^Department of Intensive Care Unit, Taihe Hospital, Hubei University of Medicine, Shiyan, China

**Keywords:** septic, corticosteroids, mortality, overlapping analysis, AMSTAR 2

## Abstract

**Background:** A host of systematic reviews and meta-analyses were carried out to estimate the role of corticosteroids in sepsis and septic shock. Discordant opinions were investigated to determine whether patients who experienced sepsis and septic shock could benefit from corticosteroids treatment. Our purpose is to perform a systematic review of overlapping meta-analyses, to explore the role of corticosteroids in the treatment of sepsis and septic shock.

**Method:** Ovid MEDLINE, EMBase, Cochrane Database of Systematic Reviews, and LILACS were searched for eligible studies. Two authors individually extracted the relevant data and evaluated the quality of the meta-analysis using A MeaSurement Tool to Assess systematic Reviews 2 (AMSTAR 2) and ROBIS. The Jadad decision algorithm was implemented to identify the meta-analyses that offered the optimal level of evidence.

**Result:** Sixteen meta-analyses met the eligibility criteria. None of the studies that reported mortality illustrated a significant improvement on mortality (14-day and 90-day), but a 28-day mortality on a long course of a low dose corticosteroids was described. Only four studies stated that a long course of low-dose corticosteroids had advantageous effect on 28-day mortality. A meta-analysis by Fang et al. was regarded as the highest level of evidence in the Jadad decision algorithm among the meta-analyses that were investigated in this systematic review.

**Conclusion:** The 28-day mortality was reduced, as well as the mortality in the ICU and hospital and the length of stay in the ICU, using a long course of low-dose corticosteroids. This was demonstrated by a meta-analysis of the current optimal available evidence. Additionally, significant improvements on the adverse events of hyperglycemia and hypernatraemia have been made.

## Introduction

Sepsis is one of the oldest and most elusive medical syndromes (Monti et al., 2015), and is a major reason for hospital mortality and critical disease (Cohen et al., 2015; Finfer and Machado, 2016). Sepsis has been reported to affect almost 300 out of 100,000 people in the USA (Cawcutt and Peters, 2014), and has resulted in over 200,000 deaths (Angus et al., 2001). The increasing mortality from sepsis and its complications have become a major public health problem worldwide and causes damage to patients regardless of their age (Suffredini and Munford, 2011). The definition of sepsis is constantly being updated since it was initially defined at a 1991 consensus conference (3), to its current definition by the Society of Critical Care Medicine (SCCM) and the European Society of Intensive Care Medicine (ESICM) (Makic and Bridges, 2018). At present, septic shock is defined as a subset of sepsis that involves aspects underlying circulatory and cellular or metabolic abnormalities, which can increase mortality triggered by a multifaceted amplified infection (Shankar-Hari et al., 2016; Singer et al., 2016; Napolitano, 2018).

Routine treatment for sepsis and septic shock include source control, fluid resuscitation, broad-spectrum antimicrobials, and vasopressors as needed (Long and Koyfman, 2017). Out of the above treatments, corticosteroids are also an important adjunctive therapy for sepsis and septic shock. Glucocorticoids are therefore the representative medicine for adjunctive therapy in patients who suffer from sepsis and septic shock, and the use of glucocorticoids attenuates the proinflammatory response, limits the anti-inflammatory response and preserves innate immunity (Marik, 2018). Exogenous glucocorticoids are extensively available given their low cost and certified capability to inhibit the inflammatory cascade (Annane et al., 2017). However, from 1976 to today, the use of corticosteroids for sepsis and septic shock has been debatable (Cawcutt and Peters, 2014; Long and Koyfman, 2017). There are two factions (salutary or detrimental effects on treatment) who are at odds over the administration of corticosteroids in sepsis and septic shock, that is, almost all studies stated that corticosteroids reduce mortality (Annane et al., 2004; Annane et al., 2009; Annane et al., 2015), while a few studies presented opposing view (Authors, 1987; Bone et al., 1987; Sprung et al., 1984; Wang et al., 2014). Additionally, meta-analyses and systematic reviews that have been published recently have suggested that a long course of low dose corticosteroids can actually save patients from sepsis and septic shock (Fang et al., 2019) (Annane et al., 2004; Annane et al., 2015). Thus, we hypothesized that a long course of low dose corticosteroids could improve the 28-day mortality of patients with sepsis and septic shock.

To investigate our hypothesis, we performed an overlapping meta-analysis aimed to elucidate the role of corticosteroids for patients with sepsis and septic shock and identified the most potent corticosteroids regimen for patient care. We also looked for evidence that gave rise to the controversial findings that have been observed for corticosteroids therapy.

## Method

### Literature Search

Several databases, including Ovid MEDLINE, EMBase, Cochrane Database of Systematic Reviews and Latin American Caribbean Health Sciences Literature (LILACS), were systematically searched for current studies that adhere to our eligibility criteria. We initiated the literature search on 20 April 2019 to identify published systematic reviews and meta-analyses. Two authors were responsible for screening the studies to obtain full manuscripts, as well as the titles and abstracts. To ensure completeness and accuracy of this review, two reviewers participated in the entire literature search process without interfering with one another. The Preferred Reporting Items for Systematic Reviews and Meta-Analyses (PRISMA) (Moher et al., 2009) was performed.

### Literature Selection and Exclusion

The inclusion criteria were as follows: 1) systematic review or meta-analysis that described relative outcome(s) associated with corticosteroids (glucocorticoids) for septic shock and sepsis, 2) a population of adults with shock and septic shock, 3) no restriction of language and publication status 4) studies must include randomized controlled trials RCTs, 5) the definitions of corticosteroids dose and course were based on included primary systematic reviews and meta-analyses.

The exclusion criteria were as follows: 1) data unavailable 2) studies involved other diseases to septic shock and septic shock and did not separate the objective data 3) a pediatric population 4) the mortality effect size was reported as a rate difference.

### Data Extraction

The following data was extracted from each study: 1) primary author, 2) date of publication, 3) date of last literature search, 4) number of included studies and included RCTs, 5) restriction of publication language and publication status, 6) search databases, 7) included primary studies 8) primary outcomes, including mortality at different days, 9) secondary outcomes, including the length of stay in the ICU and duration of hospital admission, shock reversal at day 7 and day 28, the incidence of adverse events, etc.

### Interventions

Meta-analyses and systematic reviews were used to compare treatment groups (all types of steroids such as hydrocortisone, methylprednisolone, betamethasone, fludrocortisones, dexamethasone, cortisone and other corticosteroids) with standard treatment (antibiotics, fluid replacement, inotropes, vasopressors, mechanical ventilation, renal replacement therapy) or a placebo. Moreover, when feasible, all doses and the length of administration, regardless of continuous or intermittent administration, were compared for corticosteroids.

### Assessment of Methodological Quality

To identify high quality systematic reviews, A MeaSurement Tool to Assess systematic Reviews 2 (AMSTAR 2) (Shea et al., 2017), updated in 2017, added four domains and removed one domain, resulting in the inclusion of 16 items. In contrast to departed AMSTAR (Shea et al., 2007), the AMSTAR 2 tool modified individual item ratings emphasizing the potential impact of an inadequate rating for a single item, rather than an overall score.

### Application of ROBIS

ROBIS (Whiting et al., 2016) is a tool that is used to assess the risk of bias in systematic reviews, ROBIS is completed in three phases as follows: 1) assess relevance (optional) by estimating the extent to match between target question and systematic review question, 2) identify concerns with the review process containing study eligibility criteria; identification and selection of studies; data collection and study appraisal and synthesis and findings, and 3) judge risk of bias in the review, which focused on the risk of bias caused by the conduct of reviews. This is a domain-based approach with signaling questions, following the most recent methods to assess the risk of bias.

### Heterogeneity Assessment

Heterogeneity describes an inconsistency among included studies, which is likely to affect the conclusion and result of the studies. In this overlapping meta-analysis, heterogeneity across the studies involved was tested using the I^2^ static (Higgins et al., 2003), which describes the percentage of total variation across studies that is attributable to heterogeneity rather than chance. The three levels, low, moderate, and high, were assigned according to the degree of heterogeneity *via* I^2^ values of 25%, 50%, and 75%, respectively.

### Application of Jadad Decision Algorithm

The Jadad Decision Algorithm (Jadad et al., 1997) is an adjunct decision tool for interpreting discordance among meta-analyses from the six aspects, including a clinical question, study selection and inclusion, data extraction, assessment of the study quality, and the assessment of the ability to combine studies and statistical methods for data synthesis. In general, the Jadad Decision Algorithm has been used widely for determining the current best meta-analysis or systematic reviews, by comparing populations, interventions, outcome measures, and the settings examined.

## Results

### Search Results

**Figure 1:**
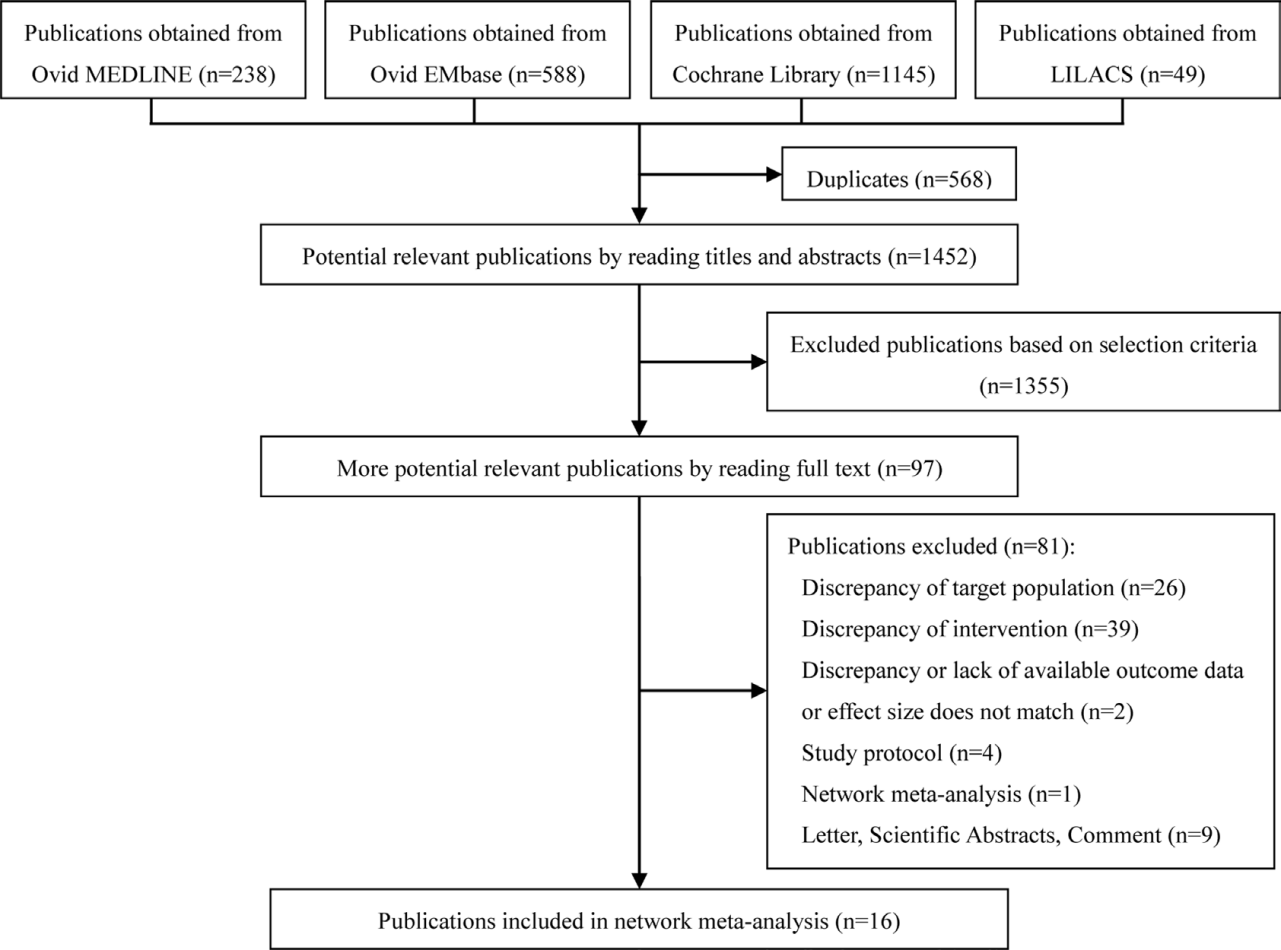
Flow diagram of searches.

The 2020 studies were found through an electrical search and only 16 studies (Annane et al., 2004; Burry and Wax, 2004; Minneci et al., 2004; Annane et al., 2009; Sligl et al., 2009; Moran et al., 2010; Wang et al., 2014; Annane et al., 2015; Volbeda et al., 2015; Lyu et al., 2018; Ni et al., 2018; Rochwerg et al., 2018; Rygard et al., 2018; Xu et al., 2018; Zhu et al., 2018; Fang et al., 2019) met the inclusion criteria in this overlapping meta-analysis. These 16 studies were published between 2004 (Annane et al., 2004) and 2019 (Lyu et al., 2018; Ni et al., 2018; Rochwerg et al., 2018; Rygard et al., 2018; Xu et al., 2018; Zhu et al., 2018; Fang et al., 2019), and recruited from 505 patients (Burry and Wax, 2004) to 10,194 patients (Rochwerg et al., 2018). Additionally, the number of primary studies varied from nine (Xu et al., 2018) to 42 (Rochwerg et al., 2018). Among these included studies, 14 studies declared no conflict of interest in their reviews (Annane et al., 2004; Minneci et al., 2004; Sligl et al., 2009; Moran et al., 2010; Wang et al., 2014; Annane et al., 2015; Volbeda et al., 2015; Lyu et al., 2018; Ni et al., 2018; Rochwerg et al., 2018; Rygard et al., 2018; Xu et al., 2018; Zhu et al., 2018; Fang et al., 2019), and only two studies were unclear about the conflict of interest (Burry and Wax, 2004; Annane et al., 2009). The details of the primary studies from the systematic reviews that were included are shown in **Supplementary Table 2**. The particular information of included systematic reviews can be found in **Table 1** and a flow diagram of the literature screen that is shown in **Figure 1**.

**Table 1:** Description of included studies.

Meta-analysis	Date of publication	Date of last literature search	No. of included studies (RCT)	Definition of Dose	Definition of Course
Annane et al., 2004	2004/08/02	2003/08	16(15)	High dose: ≥300 mg/day; Low dose: <300 mg/day	Short course: <5 days; Long course: ≥5 days
Burry and Wax, 2004	2004/01/23	2003/03	6(6)	Low dose: 200–300 mg^#^	NR
Minneci et al., 2004	2004/07	2003/12	14(14)	Unclear	Unclear
Annane et al., 2009	2009/06/10	2009/03	22*(17)	High dose: ≥300 mg/day; Low dose: <300 mg/day	Short course n: <5 days; Long course: ≥5 days
Sligl et al., 2009	2009/06/2	2008/12	8(6)	Unclear	Unclear
Moran et al., 2010	2010/07/13	2008/09	14(14)	High dose: ≥1,000 mg/day; Low dose: <1,000 mg/day	NR
Wang et al., 2014	2014/02	2012/05	8(8)	High dose: ≥300 mg/day; Low dose: <300 mg/day	Short course: <5 days; Long course: ≥5 days
Volbeda et al., 2015	2015/06/23	2015/02/18	35(35)	High dose: ≥500 mg/day; Low dose: <500 mg/day	NR
Annane et al., 2015	2015	2014/10	33(33)	High dose: ≥400 mg/day; Low dose: <400 mg/day	Short course: <3 days; Long course: ≥3 days
Rochwerg et al., 2018	2018	2018/01/10	42(42)	High dose: ≥400 mg/day; Low dose: <400 mg/day	Short course: <3 days; Long course: ≥3 days
Rygard et al., 2018	2018	2018/03/03	22(22)	High dose: ≥500 mg/day; Low dose: <500 mg/day	NR
Xu et al., 2018	2018/04/10	2017/10/23	9(9)	High dose: ≥500 mg/day; Low dose: <500 mg/day	Short course: <3 days; Long course: ≥3 days
Zhu et al., 2018	2018	2018/03/24	18(18)	Unclear	Unclear
Fang et al., 2019	2018/12/21	2018/08/10	37(37)	High dose: ≥400 mg/day; Low dose: <400 mg/day	Short course: <4 days; Long course: ≥4 days
Lyu et al., 2018	2018/09/5	2018/03/07	13(13)	High dose: ≥200 mg/day; Low dose: <200 mg/day	NR
Ni et al., 2018	2018/11/26	2018/03	19(19)	High dose: ≥300 mg/day; Low dose: <300 mg/day	NR

RCTs, Randomized controlled trials; NR, Not reporte, Unclear, The definitions of dose or course were not reported in systematic reviews and meta-analyses, *indicates that including 17 RCTs, 3 quasi-randomized trials and 2 conference proceedings. ^#^short-term, high-dose intravenous methylprednisolone (30 mg/kg every 6 h for 4 doses or a30-mg/kg bolus followed by a 5-mg/kg/h infusion for 9 h).

### Search Methodology

Confining to English language was reported in only two studies (Sligl et al., 2009; Moran et al., 2010), and four studies (Minneci et al., 2004; Sligl et al., 2009; Moran et al., 2010; Zhu et al., 2018) reported that their the publication status was restricted as published studies. All the studies (Annane et al., 2004; Burry and Wax, 2004; Minneci et al., 2004; Annane et al., 2009; Sligl et al., 2009; Moran et al., 2010; Wang et al., 2014; Annane et al., 2015; Volbeda et al., 2015; Lyu et al., 2018; Ni et al., 2018; Rochwerg et al., 2018; Rygard et al., 2018; Xu et al., 2018; Zhu et al., 2018; Fang et al., 2019) used PubMed/Medline, and both EMBase and Cochrane Central Register of Controlled Trials (CENTRAL). The Cochrane Library to search literature was used by all studies except Minneci et al (Minneci et al., 2004), LILACS was used by five meta-analyses (Annane et al., 2004; Annane et al., 2009; Wang et al., 2014; Annane et al., 2015; Rochwerg et al., 2018), however, only four studies (Annane et al., 2004; Annane et al., 2009; Wang et al., 2014; Annane et al., 2015) searched the Cochrane infectious diseases group’s trial register. The electronic literature search strategies are shown in detail in **Supplementary Table 1**.

### Outcome Measures

The primary outcome chiefly included mortality at day 28, especially for mortality due to a long course of low dose corticosteroids, as well as 90-day mortality. Mortality at 30 days was classified as 28-day mortality in this review, since only one study reported this (Volbeda et al., 2015). A mortality subgroup analysis was preformed according to the number of days, ranked in ascending order (14 days to 1 year), and different courses with diverse doses. In addition, the mortality of ICU and hospital admissions, the length of stay in the ICU and hospital, and adverse events and shock reversal, were investigated as secondary outcomes. The length of stay in the ICU and hospital and shock reversal on day 7 or day 28, also served a crucial role in assessing the benefit of corticosteroids in treating septic shock and sepsis in recent years. Meanwhile, we cannot ignore corticosteroids-associated adverse events, which involve gastroduodenal bleeding or gastro-intestinal bleeding, superinfection or secondary infections, hyperglycemia, hypernatraemia, neuromuscular weakness, and so on. All of the outcomes of each meta-analysis or systematic review can be found in **Supplementary Tables 3** and **4**.

### Study Quality and Validity

AMSTAR 2—an updated appraisal instrument for high quality meta-analyses, underlines critical domains rather than the total score, and the details of individual studies are presented in **Table 2**. We investigated the overall rating confidence of the results of each review using a checklist from the AMSTAR website (www.amstar.ca). Meanwhile, the quality of each meat-analysis and systematic review was examined using the AMSTAR 2 tool. We found that the study by Fang et al met the 14 items of the AMSTAR instrument and most likely offered the most comprehensive meta-analysis of the role of corticosteroids in sepsis and septic shock. ROBIS endorses domain-based approaches for integral systematic reviews in a similar manner to AMSTAR 2, and judges risk of bias in the review process, results and conclusion as “low,” “high,” or “unclear,” and guides recommendations to improve patient care. These results are presented in a tabular graph (**Table 3**).

**Table 2:** Application of AMSTAR 2.

Items of AMSTAR 2	Annane et al., 2004	Burry and Wax, 2004	Minneci et al., 2004	Annane et al., 2009	Sligl et al., 2009	Moran et al., 2010	Wang et al., 2014	Volbeda et al., 2015	Annane et al., 2015	Rochwerg et al., 2018	Rygard et al., 2018	Xu et al., 2018	Zhu et al., 2018	Fang, et al., 2019	Lyu et al., 2018	Ni et al., 2018
1. Did the research questions and inclusion criteria for the review include the components of PICO?	Y	Y	Y	Y	Y	Y	Y	Y	Y	Y	Y	Y	Y	Y	Y	Y
2. Did the report of the review contain an explicit statement that the review methods were established prior to the conduct of the review and did the report justify any significant deviations from the protocol?	P	P	Y	Y	P	P	P	Y	Y	Y	Y	Y	P	Y	Y	P
3. Did the review authors explain their selection of the study designs for inclusion in the review?	Y	U	U	Y	U	Y	U	N	Y	U	Y	U	Y	Y	P	Y
4. Did the review authors use a comprehensive literature search strategy?	Y	Y	P	Y	Y	Y	Y	Y	Y	Y	Y	Y	Y	Y	Y	Y
5. Did the review authors perform study selection in duplicate?	Y	Y	Y	Y	Y	Y	Y	Y	Y	Y	Y	Y	Y	Y	Y	Y
6. Did the review authors perform data extraction in duplicate?	Y	Y	Y	Y	Y	Y	Y	Y	Y	Y	Y	Y	Y	Y	Y	Y
7. Did the review authors provide a list of excluded studies and justify the exclusions?	Y	N	N	Y	Y	Y	Y	Y	Y	N	N	N	N	N	N	N
8. Did the review authors describe the included studies in adequate detail?	Y	N	Y	Y	P	Y	P	Y	Y	P	P	P	Y	Y	Y	Y
9. Did the review authors use a satisfactory technique for assessing the risk of bias (RoB) in individual studies that were included in the review?	Y	Y	Y	Y	Y	Y	Y	Y	Y	Y	Y	Y	Y	Y	Y	Y
10. Did the review authors report on the sources of funding for the studies included in the review?	N	N	N	N	N	N	N	N	N	N	N	N	N	N	N	N
11. If meta-analysis was performed, did the review authors use appropriate methods for statistical combination of results?	Y	Y	Y	Y	Y	Y	Y	Y	Y	Y	Y	Y	Y	Y	Y	Y
12. If meta-analysis was performed, did the review authors assess the potential impact of RoB in individual studies on the results of the meta-analysis or other evidence synthesis?	Y	Y	Y	Y	Y	Y	Y	Y	Y	Y	Y	Y	Y	Y	Y	Y
13. Did the review authors account for RoB in primary studies when interpreting/discussing the results of the review?	N	N	N	Y	N	N	Y	Y	Y	Y	Y	Y	Y	N	Y	Y
14. Did the review authors provide a satisfactory explanation for, and discussion of, any heterogeneity observed in the results of the review?	Y	N	Y	Y	Y	Y	Y	Y	Y	Y	Y	Y	Y	Y	Y	Y
15. If they performed quantitative synthesis did the review authors carry out an adequate investigation of publication bias (small study bias) and discuss its likely impact on the results of the review?	Y	N	N	Y	Y	Y	Y	Y	Y	Y	Y	Y	Y	Y	N	Y
16. Did the review authors report any potential sources of conflict of interest, including any funding they received for conducting the review?	Y	U	Y	U	Y	Y	Y	Y	Y	Y	Y	Y	Y	Y	Y	Y

AMSTAR 2, A MeaSurement Tool to Assess systematic Reviews; Y, Yes; N, No; P, Partial Yes; U, Unclear.

**Table 3:** The result of ROBIS.

Meta-analysis	Phase 2				Phase 3 Risk of bias in the review
	1. Study eligibility criteria	2. Identification and selection of studies	3. Data collection and study appraisal	4. Synthesis and findings	
Annane et al., 2004	☹	☺	☺	☹	☺
Burry and Wax, 2004	☹	☹	☹	☹	☹
Minneci et al., 2004	☹	☹	☹	☹	☹
Annane et al., 2009	☹	☺	☺	☹	☺
Sligl et al., 2009	☹	☹	☹	☹	☹
Moran et al., 2010	☹	☹	☺	☹	☺
Wang et al., 2014	☹	☺	☹	☹	☹
Volbeda et al., 2015	☺	☺	☺	☺	☹
Annane et al., 2015	☺	☺	☺	☺	☺
Rochwerg et al., 2018	☺	☺	☺	☺	☺
Rygard et al., 2018	☺	☹	☺	☺	☺
Xu et al., 2018	☺	☹	☺	☹	☹
Zhu et al., 2018	☹	☹	☺	☹	☹
Fang et al., 2019	☺	☺	☺	☺	☺
Lyu et al., 2018	☹	☺	☺	☺	☺
Ni et al., 2018	☹	☹	☺	☺	☺

☹ = low risk; ☺ = high risk.

### Heterogeneity Assessment

Of the 16 systematic reviews and meta-analyses that were included in this overlapping review (Annane et al., 2004; Burry and Wax, 2004; Minneci et al., 2004; Annane et al., 2009; Sligl et al., 2009; Moran et al., 2010; Wang et al., 2014; Volbeda et al., 2015; Annane et al., 2015; Lyu et al., 2018; Ni et al., 2018; Rochwerg et al., 2018; Rygard et al., 2018; Xu et al., 2018; Zhu et al., 2018; Fang et al., 2019), 15 studies (Annane et al., 2004; Minneci et al., 2004; Annane et al., 2009; Sligl et al., 2009; Moran et al., 2010; Wang et al., 2014; Annane et al., 2015; Volbeda et al., 2015; Lyu et al., 2018; Ni et al., 2018; Rochwerg et al., 2018; Rygard et al., 2018; Xu et al., 2018; Zhu et al., 2018; Fang et al., 2019) performed and reported statistical heterogeneity analysis, and only one study (Burry and Wax, 2004) discussed, but did not analyze, descriptive data. All of the 17 studies (Annane et al., 2004; Burry and Wax, 2004; Minneci et al., 2004; Annane et al., 2009; Sligl et al., 2009; Moran et al., 2010; Wang et al., 2014; Annane et al., 2015; Volbeda et al., 2015; Lyu et al., 2018; Ni et al., 2018; Rochwerg et al., 2018; Rygard et al., 2018; Xu et al., 2018; Zhu et al., 2018; Fang et al., 2019) carried out primary study quality and showed the relevant data from individual studies. With respect to the size of the primary studies, Seven studies (Minneci et al., 2004; Moran et al., 2010; Wang et al., 2014; Lyu et al., 2018; Ni et al., 2018; Zhu et al., 2018; Fang et al., 2019) reported and analyzed this parameter; three systematic reviews (Burry and Wax, 2004; Annane et al., 2015; Volbeda et al., 2015) discussed it, but did not present concrete data, while the rest of systematic reviews (Annane et al., 2004; Annane et al., 2009; Sligl et al., 2009; Rochwerg et al., 2018; Rygard et al., 2018; Xu et al., 2018) did not conduct a formal sensitivity or subgroup analysis. With respect to publication bias, 14 studies (Annane et al., 2004; Annane et al., 2009; Sligl et al., 2009; Moran et al., 2010; Wang et al., 2014; Annane et al., 2015; Volbeda et al., 2015; Lyu et al., 2018; Ni et al., 2018; Rochwerg et al., 2018; Rygard et al., 2018; Xu et al., 2018; Zhu et al., 2018; Fang et al., 2019) executed the analysis, but two studies (Burry and Wax, 2004; Minneci et al., 2004) did not. As for the result of other parameters on outcomes such as the duration and/or dose of corticosteroids, the mortality on different days, the mortality of ICU after receiving different dose, etc., are presented in **Supplementary Table 5**.

## Study Result

### Primary Outcome

**Figure 2:**
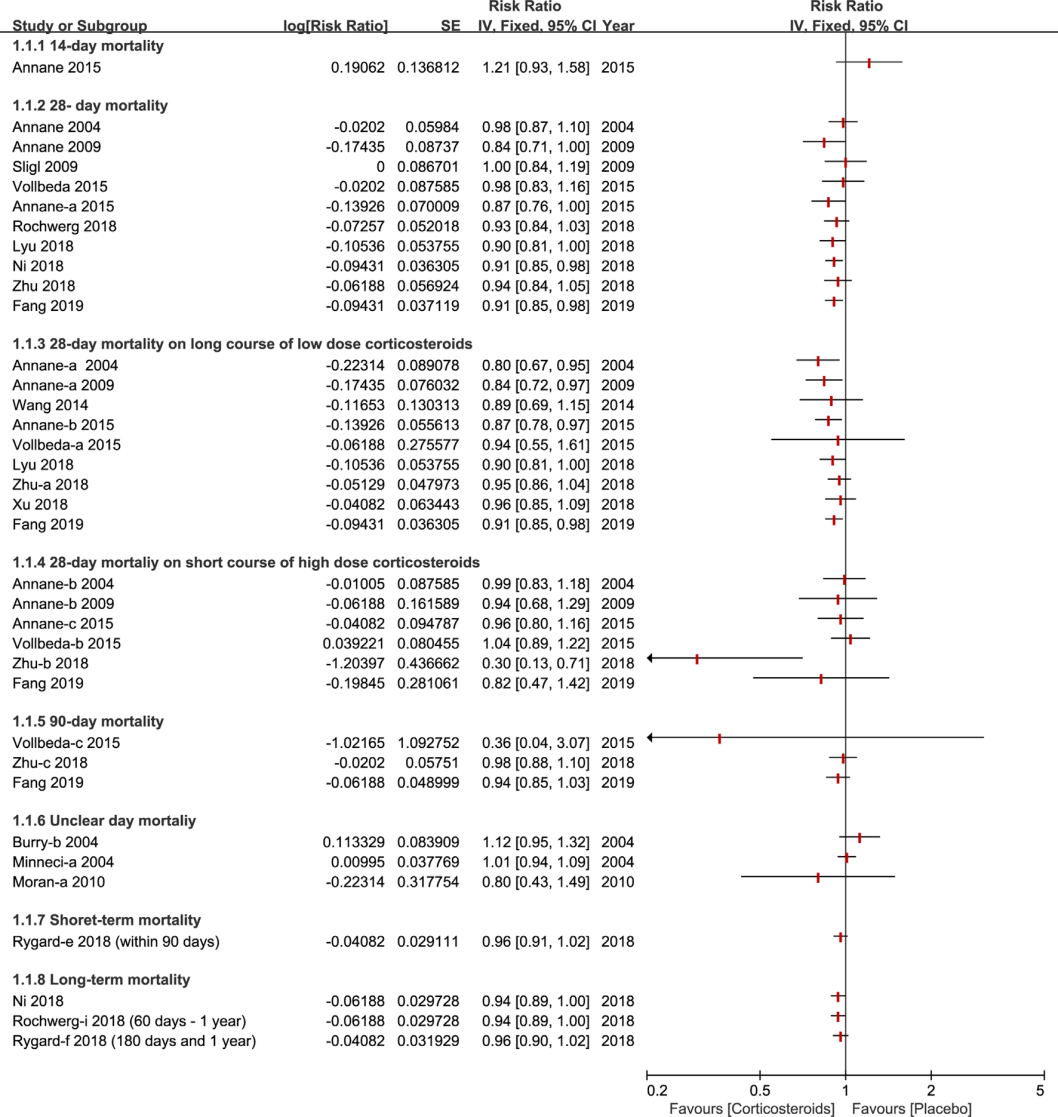
Forest plot of mortality according to days ranked in ascending order (fourteen days to one year) with different courses in diverse dose (long course of low dose corticosteroids and short course of high dose corticosteroids).

Almost all of studies included a 28-day mortality after corticosteroids therapy (Annane et al., 2004; Burry and Wax, 2004; Minneci et al., 2004; Annane et al., 2009; Sligl et al., 2009; Moran et al., 2010; Wang et al., 2014; Annane et al., 2015; Lyu et al., 2018; Ni et al., 2018; Rochwerg et al., 2018; Rygard et al., 2018; Xu et al., 2018; Zhu et al., 2018; Fang et al., 2019). Different viewpoints were presented across different meta-analyses about whether different doses of corticosteroids (especially on long course of low dose corticosteroids) could decrease the 28-day mortality. A couple of studies found that 28-day all-cause mortality didn’t show a significant effect, and a high or low dose of corticosteroids for sepsis or septic shock, did not show a significant effect either (Sligl et al., 2009; Volbeda et al., 2015; Lyu et al., 2018; Ni et al., 2018; Rochwerg et al., 2018; Zhu et al., 2018), while others held the opposite view (Annane et al., 2004; Annane et al., 2009; Annane et al., 2015; Fang et al., 2019). Ninety-day mortality was also listed as primary outcomes, in which two studies presented similar data (Zhu et al., 2018; Fang et al., 2019), while three studies offered relevant data (Volbeda et al., 2015; Zhu et al., 2018; Fang et al., 2019). Furthermore, short-term and long-term mortality were divided based on the different standards of three studies (Rochwerg et al., 2018; Rygard et al., 2018; Fang et al., 2019), all published in 2018 and 2019. The details of mortality based on different days are shown in **Figure 2**.

### Mortality of Hospital and ICU

**Figure 3:**
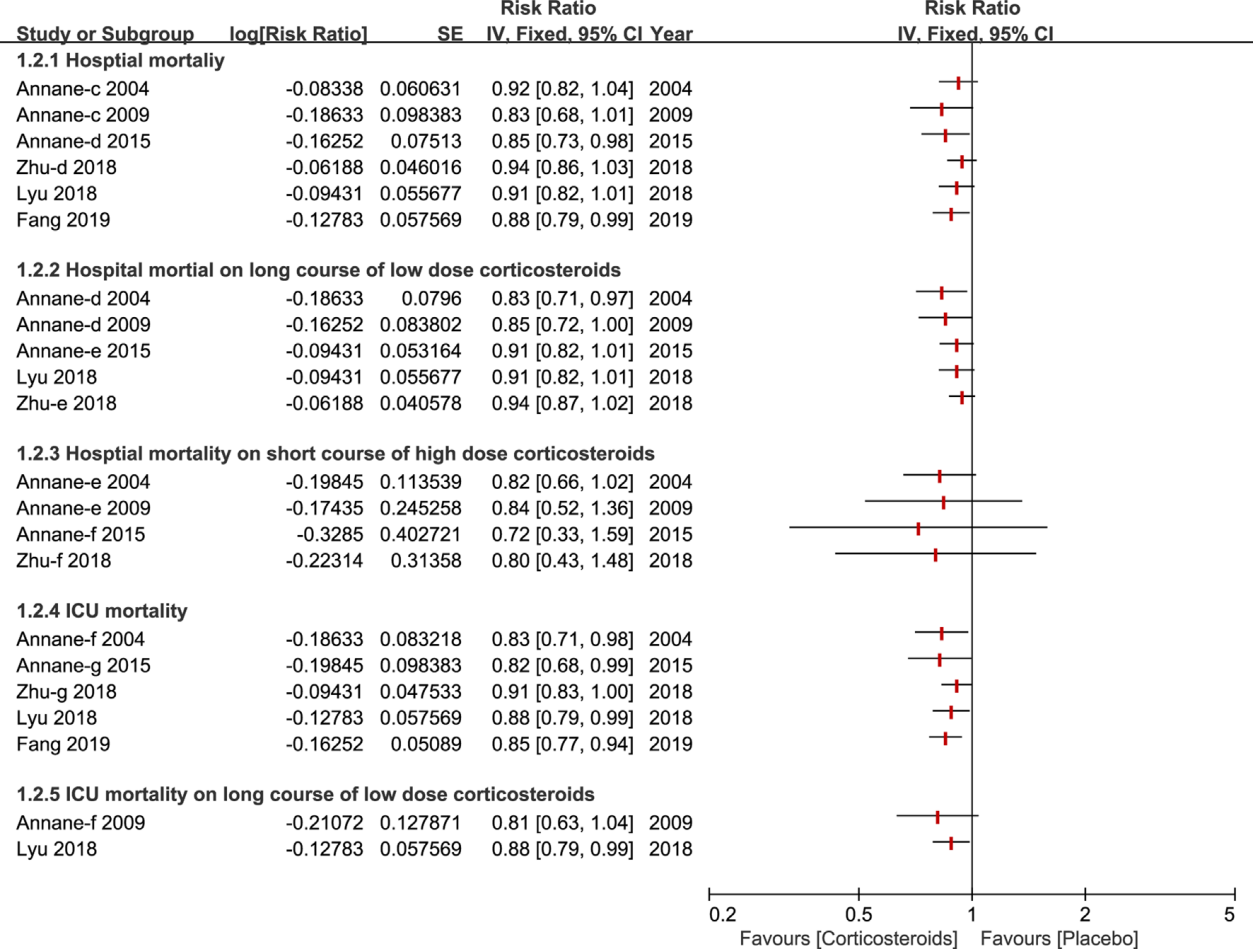
Forest plot of mortality of ICU and Hospital by subgroup analysis based on long course of low dose corticosteroids and short course of high dose corticosteroids.

A total of six studies (Annane et al., 2004; Annane et al., 2009; Annane et al., 2015; Lyu et al., 2018; Zhu et al., 2018; Fang et al., 2019) reported the outcome of hospital mortality and of those, three studies (Annane et al., 2004; Annane et al., 2009; Zhu et al., 2018) demonstrated no significant impact on hospital mortality using corticosteroids for patients with sepsis and septic shock. All of these studies performed a subgroup analysis based on duration and dose, and the results of this outcome are described in **Figure 3**. Additionally, six studies paid particular attention to ICU mortality (Annane et al., 2004; Annane et al., 2009; Annane et al., 2015; Lyu et al., 2018; Zhu et al., 2018; Fang et al., 2019), and two (Annane et al., 2009; Lyu et al., 2018) of the six studies kept an eye on ICU mortality on a long course of low-dose corticosteroids.

### Length of Stay in ICU and Hospital

**Figure 4:**
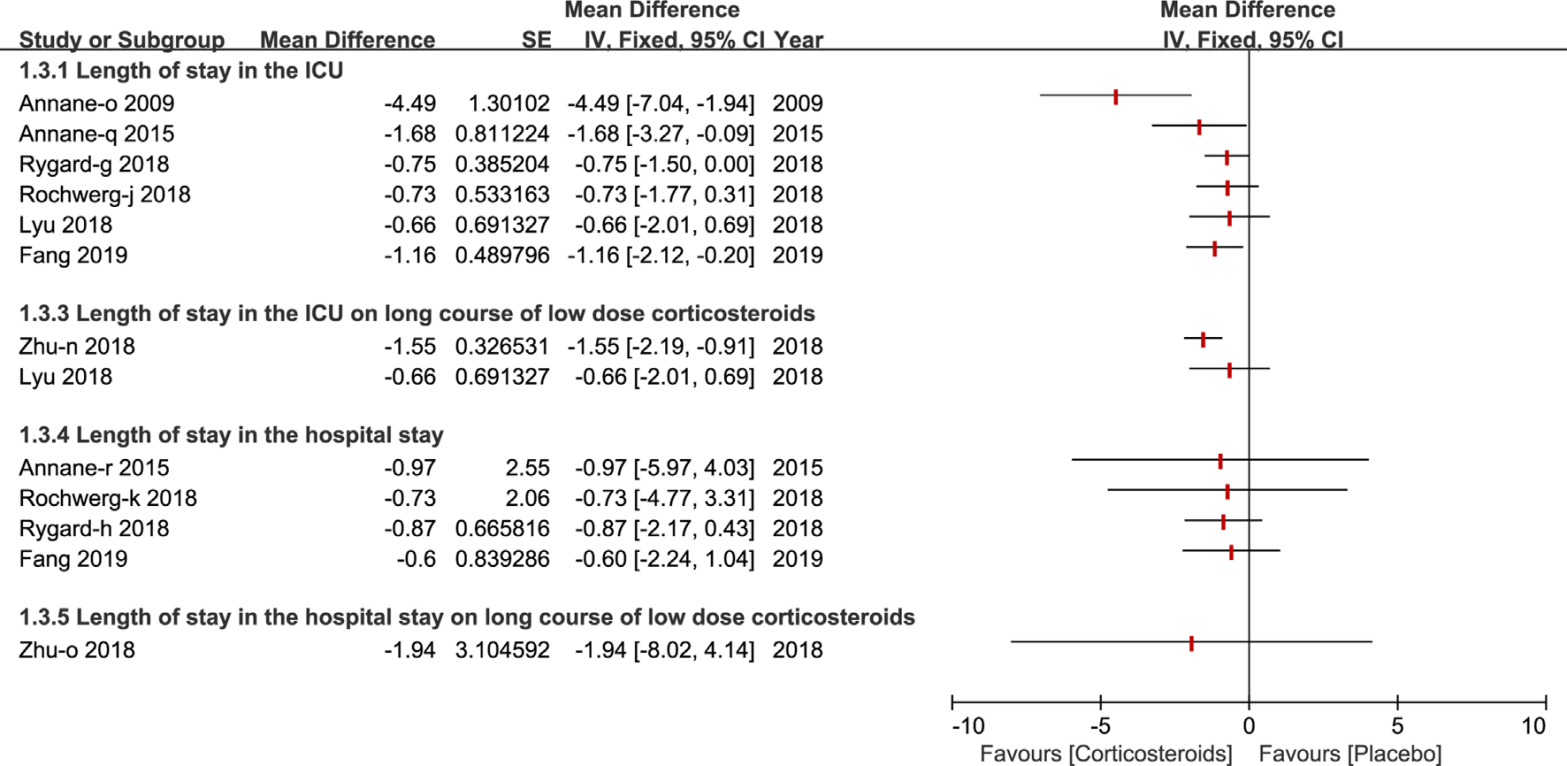
Forest plot of the length of patients stay in ICU and hospital by subgroup analysis based on long course of low dose corticosteroids and short course of high dose corticosteroids.

The length of stay in the ICU was reported by six studies (Annane et al., 2009; Annane et al., 2015; Lyu et al., 2018; Rochwerg et al., 2018; Rygard et al., 2018; Fang et al., 2019) and three studies reported the length of stay in the hospital (Annane et al., 2015; Rochwerg et al., 2018; Rygard et al., 2018). Only a few studies published in 2018 and 2019 carried out a subgroup analysis on a long course of low dose corticosteroids (Zhu et al., 2018; Fang et al., 2019) (see **Figure 4**).

### Shock Reversal

**Figure 5:**
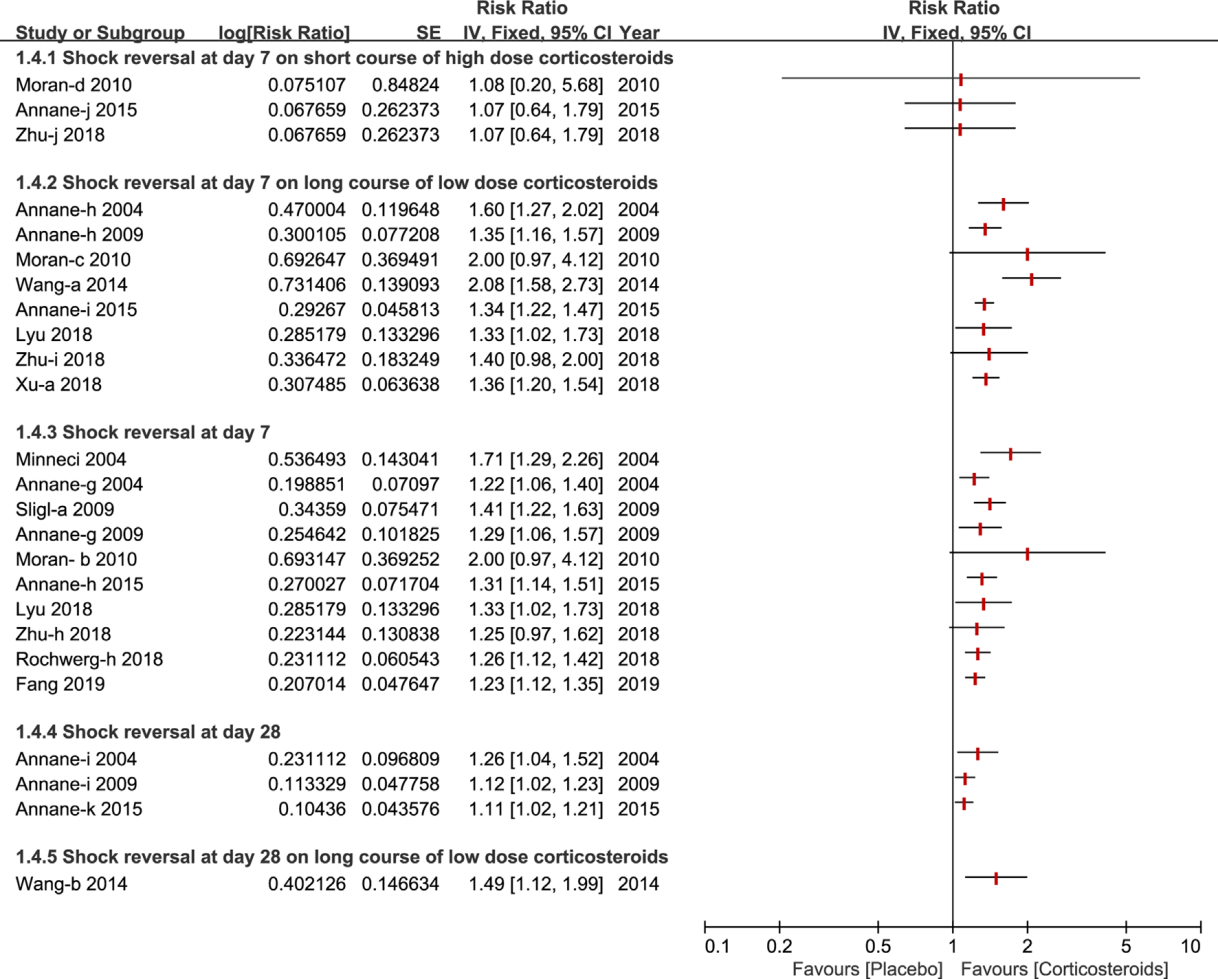
Forest plot of reversal of shock at day 7 and day 28 by subgroup analysis based on long course of low dose corticosteroids and short course of high dose corticosteroids.

Shock reversal was stratified into two cohorts, shock reversal at day 7 and day 28. Twelve studies compared a treatment group (corticosteroids treatment) and control group (placebo or standard treatment)(Annane et al., 2004; Minneci et al., 2004; Annane et al., 2009; Sligl et al., 2009; Moran et al., 2010; Wang et al., 2014; Annane et al., 2015; Lyu et al., 2018; Rochwerg et al., 2018; Xu et al., 2018; Zhu et al., 2018; Fang et al., 2019). Shock reversal at day 7 was reported by nine studies (Annane et al., 2004; Minneci et al., 2004; Annane et al., 2009; Sligl et al., 2009; Moran et al., 2010; Annane et al., 2015; Rochwerg et al., 2018; Zhu et al., 2018; Fang et al., 2019). Seven of the nine studies performed a further subgroup analysis of a long course of low-dose corticosteroids, and found that low-dose corticosteroids over a long course, contributed to shock reversal at day 7 (Annane et al., 2004; Annane et al., 2009; Moran et al., 2010; Wang et al., 2014; Annane et al., 2015; Xu et al., 2018; Zhu et al., 2018). Not many studies reported a short course of high-dose corticosteroids (Moran et al., 2010; Annane et al., 2015; Zhu et al., 2018). As such, Wang et al (Wang et al., 2014) presented a subgroup analysis of shock reversal at day 28 for a long course of low dose corticosteroids. All of details about the shock reversal are illustrated in **Figure 5**.

### Adverse Events

**Figure 6:**
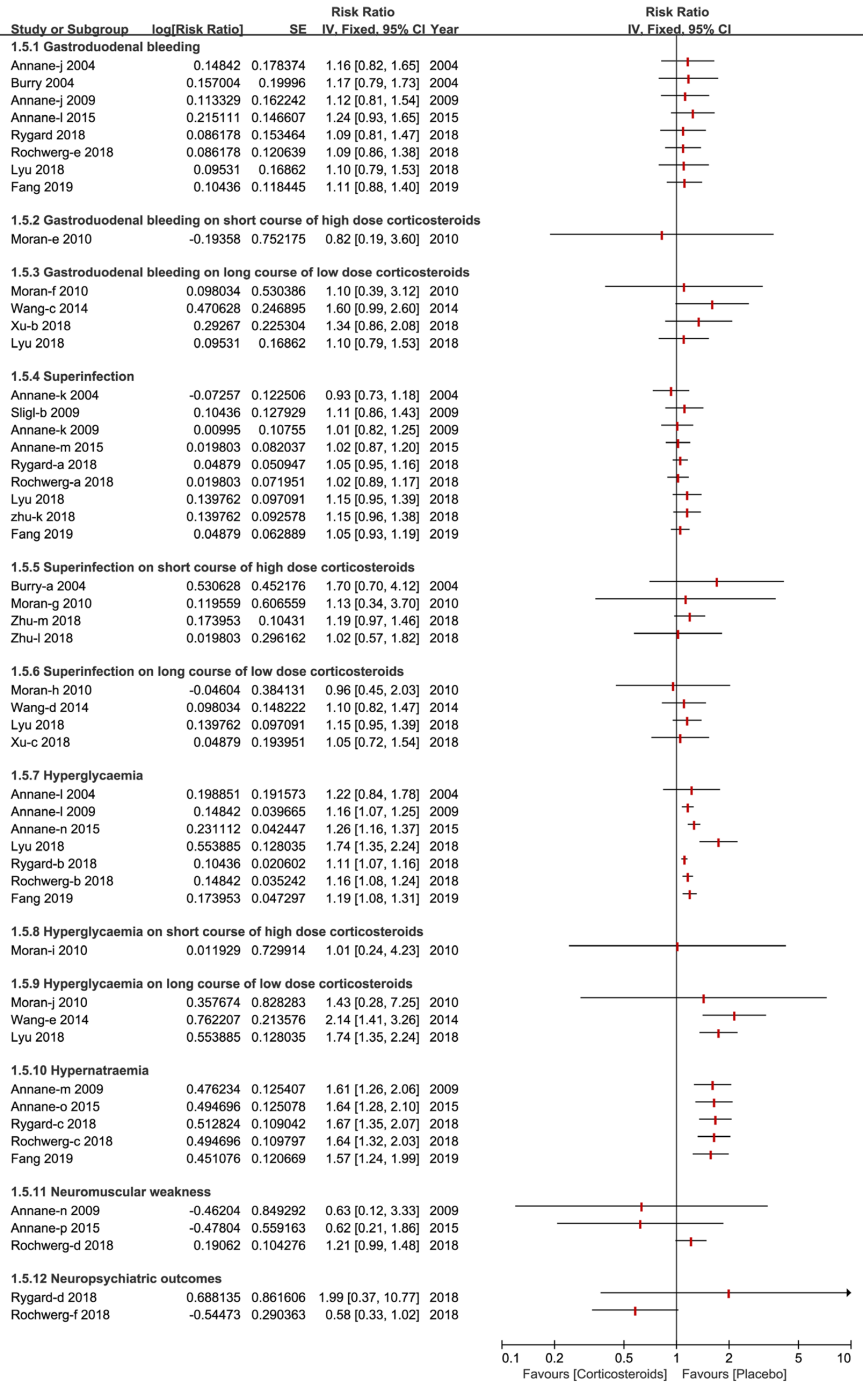
Forest plot of adverse events by subgroup analysis based on long course of low dose corticosteroids and short course of high dose corticosteroids.

A total of 10 types of adverse events associated with corticosteroids were analyzed by the included studies in this overlapping meta-analysis. Gastroduodenal bleeding, superinfection, hyperglycemia and hypernatraemia were the most common adverse events and were reported and discussed by almost all of the studies included. Other outcomes of the adverse events reported, are fully described in **Figure 6**.

### Application of Jadad Decision Algorithm

**Figure 7:**
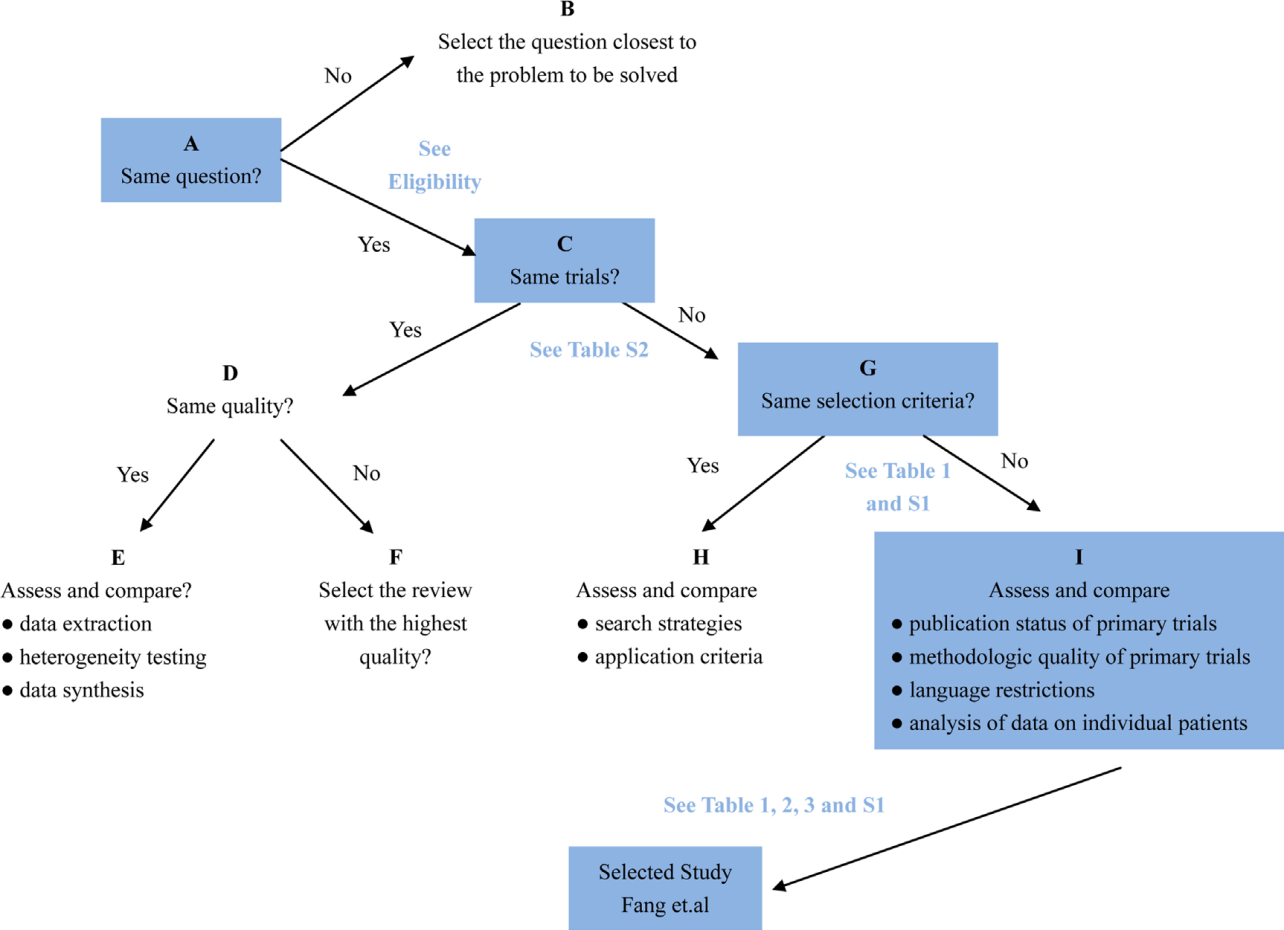
Flow diagram of Jadad decision algorithm showed process and selection of the highest level of evidence across included meta-analyses and systematic reviews.

The Jadad Decision Algorithm was applied to identify which meta-analyses offered the best evidence among the included studies and was dependently implemented by two lead authors. A meta-analysis provided the highest level of currently available evidence (Fang et al., 2019), based on the Jadad Decision Algorithm, and found that patients with septic shock or sepsis who used a long course of low dose corticosteroids tended to benefit, as manifested by a decreased mortality of 28 days (**Figure 7**).

## Discussion

Whether corticosteroids have an effect on sepsis and septic shock remains unclear and a definite conclusion has not been achieved. This overlapping meta-analysis probed the efficacy and safety of corticosteroids for sepsis and septic shock and sought to identify the reason for discordance across the various meta-analyses, as the most comprehensive overlapping meta-analysis performed to date. Multiple studies, that included meta-analyses and systematic reviews (Annane et al., 2004; Burry and Wax, 2004; Minneci et al., 2004; Annane et al., 2009; Sligl et al., 2009; Moran et al., 2010; Wang et al., 2014; Annane et al., 2015; Volbeda et al., 2015; Rochwerg et al., 2018; Rygard et al., 2018; Xu et al., 2018; Zhu et al., 2018) and primary studies (Cohen et al., 2015; Annane et al., 2017; Long and Koyfman, 2017; Annane et al., 2018; Makic and Bridges, 2018), were explored this investigation. A detailed elaboration of diverse doses and length of corticosteroids administration for patients who suffer from sepsis and septic shock was investigated in a meta-analysis by Fang et al. We selected the current best meta-analyses and systematic reviews using several methodologic appraisal tools (AMSTAR 2, ROBIS, and Jadad decision algorithm). Using these assessment tools, we found the meta-analysis with the highest and best level of evidence (Fang et al., 2019).

There are 16 studies included in this article (Annane et al., 2004; Burry and Wax, 2004; Minneci et al., 2004; Annane et al., 2009; Sligl et al., 2009; Wang et al., 2014; Volbeda et al., 2015; Moran et al., 2010; Annane et al., 2015; Lyu et al., 2018; Ni et al., 2018; Rochwerg et al., 2018; Rygard et al., 2018; Xu et al., 2018; Zhu et al., 2018; Fang et al., 2019), almost all of which reported 28-day mortality and discussed this outcome. The key study by Fang et al compared a long course of low dose corticosteroids and a short course of high dose corticosteroids and concluded that the 28-day mortality rate improved after the administration of a long course of low-dose corticosteroids. Moreover, the 28-day mortality after a long courses of low dose corticosteroids, has shown discrepancies among the four meta-analyses that were published in 2018 and 2019 (Lyu et al., 2018; Xu et al., 2018; Zhu et al., 2018; Fang et al., 2019). It all boils down to two respects that included participant characteristic and included primary studies. A 90-day mortality appeals to more researchers, and it might be vital evidence to reflect the validity of applying a long course of low dose corticosteroids for sepsis and septic shock. Recent reviews of corticosteroids have reached a consensus that a 90-day mortality had no improvement for patients who received treatment of a long course of low dose corticosteroids (Volbeda et al., 2015; Zhu et al., 2018; Fang et al., 2019).

With respect to the terms of mortality, a 90-day mortality cut-off point by was used by Rygard et al (Rygard et al., 2018) and Fang et al (Fang et al., 2019). Different to Rygard et al (Rygard et al., 2018) and Fang et al (Fang et al., 2019), Rochwerg et al (Rochwerg et al., 2018) investigated 60 days to 1 year mortality as a long term mortality and marked 28-day to 31-day mortality as a short term mortality caused by sepsis and septic shock.

In contrast to other studies that reported hospital mortality, Annane et al. (2015), Fang et al. (2019) and Lyu et al. (2018) provided data that favors corticosteroids. The study with the current best available evidence demonstrated that a long course of low-dose corticosteroids could improve ICU mortality (Fang et al., 2019). Shock reversal was one a beneficial outcome, occurring as early as 2004, by Annane et al. (2004). Eight studies presented the idea that shock reversal at day 7 could benefit from the treatment of corticosteroids (Annane et al., 2004; Minneci et al., 2004; Annane et al., 2009; Sligl et al., 2009; Moran et al., 2010; Annane et al., 2015; Rochwerg et al., 2018; Zhu et al., 2018). Furthermore, three studies by Annane et al published in 2004, 2009 and 2015 reported the reversal of shock at day 28, which displayed a positive effect that favors corticosteroids (Annane et al., 2004; Annane et al., 2009; Annane et al., 2015).

The length of stay in the ICU and hospital also reflect the benefits of corticosteroids for patients who develop sepsis and septic shock. A recent meta-analyses of corticosteroids disclosed the details of results of the length of stay in the ICU and hospital, and the findings of these studies were so divergent that we could ultimately draw the conclusion that corticosteroids have no immediate benefit on the length of stay in the hospital (Annane et al., 2009; Annane et al., 2015; Lyu et al., 2018; Rochwerg et al., 2018; Rygard et al., 2018; Zhu et al., 2018) but benefit the length of stay in the ICU (Annane et al., 2009; Annane et al., 2015; Fang et al., 2019). The severity of adverse events is likely to increase mortality and affects the application of corticosteroids for participants with sepsis and septic shock. We found that regardless of the dose and course duration of corticosteroids, the incidence of gastroduodenal bleeding did not significantly increase. Nine studies elaborated no striking impact on superinfection or secondary infection apropos of high dose corticosteroids, as well as a long course of low dose corticosteroids (Annane et al., 2004; Annane et al., 2009; Sligl et al., 2009; Annane et al., 2015; Lyu et al., 2018; Rochwerg et al., 2018; Rygard et al., 2018; Zhu et al., 2018; Fang et al., 2019).

At present, the guidelines of sepsis management that were updated in 2013 advocate that corticosteroids that are used in the context of adequate fluid resuscitation and vasopressor treatment, have the capability of restoring hemodynamic stability (Dellinger et al., 2013). The newest guidelines by Rhodes et al further demonstrated that both agreed with hydrocortisone therapy at 200 mg per day (Rhodes et al., 2017).

## Limitations

The originality and importance of this overlapping meta-analysis is with respect to its exploration of comprehensive outcomes to determine whether corticoid steroid therapy is harmful or beneficial for patients with sepsis and septic shock. We provide insights into the existing differences among various meta-analyses and systematic reviews and offer relevant suggestions about strategies for using corticosteroids. Additionally, we used the newest methodologic assessment tools including AMSTAR 2 and ROBIS, which were published in 2017 and 2016, respectively. These could better comprehensively assess the meta-analyses and systematic reviews included, acquiring the current best available evidence. The 28-day mortality with the use of a long course of low-dose corticosteroids was in the list of critical aspects and was discussed and analyzed. In addition, we conducted heterogeneity and subgroup analyses of the primary studies to better reveal the sources of heterogeneity from the primary studies. We only included 16 systematic reviews and meta-analyses that met our eligibility criteria and hope that more studies can be included in the future. Only RCTs were included in this study. Furthermore, there was insufficient data of mortality on 90 days or longer periods, owing to the fact that fewer studies reported these statistics. Understanding the link between corticosteroids, sepsis and septic shock will help clinical staff and decision-makers in ensuring optimal care for patients who receive long courses of low-dose corticosteroid therapy.

The following aspects need to be investigated in the future: (1) First, the accurate dose for distinguishing high and low doses, as well as short- and long-courses, has not been resolved at present. The included studies have their own classification standards about doses and terms. (2) As such, the optimal strategy of involving corticosteroids dosage and preferred glucocorticoid remains ambiguous. These issues could have influenced the result of corticosteroids for sepsis and sepsis shock.

## Conclusion

A comparison of the findings across studies has allowed us to confirm that a long course of low-dose corticosteroids contribute in reducing 28-day mortality, mortality of ICU and hospital stays and the length of stay in the ICU for patients undergoing therapy for sepsis and septic shock. However, no improvement was found in long-term mortality outcomes, such as 90-day mortality. Concerning adverse events, except for hyperglycemia and hypernatraemia, no other significant improvement was observed.

## Author Contributions

CZ and Y-MN conceptualized and coordinated the study. CZ drafted the initial protocol. Y-MN developed the search strategy. L-LL, Y-YY, and H-YG screened citations and assessed studies for eligibility. L-LL and Y-YY extracted data. H-YG and J-YW performed quality assessments. Y-YY and J-YW provided content expertise in corticosteroids and Sepsis/Septic Shock. CZ and Y-MN provided methodologic expertise in knowledge synthesis and resolved disagreements regarding study eligibility or quality assessments. CZ, Y-MN, and L-LL critically reviewed the manuscript for important intellectual content. All of the authors provided final approval of the version to be published and agreed to be accountable for all aspects of the work.

## Conflict of Interest Statement

The authors declare that the research was conducted in the absence of any commercial or financial relationships that could be construed as a potential conflict of interest.
