# Supplementary material for: Are Corticosteroids Beneficial for Sepsis and Septic Shock? Based on Pooling Analysis of 16 Studies
Source: Front Pharmacol. 2019 Jul 12;10:714. doi: 10.3389/fphar.2019.00714 (PMC6640176; doi:10.3389/fphar.2019.00714)
Supplement: Supplementary file 1 [file Table_1.docx]

**SUPPLEMENTAL MATERIAL**

**Supplementary Table 1** Search methodology used by each included study

**Supplementary Table 2** Primary studies included in meta-analysis

**Supplementary Table 3** Outcomes reported by individual included study

**Supplementary Table 4** Adverse events included in meta-analysis

**Supplementary Table 5** Heterogeneity or subgroup analyses of primary studies

**Supplementary Table 1** Search methodology used by each included study

| **Meta-analysis** | **Restriction of publication language** | **Restriction of publication status** | **Search databases** | | | | | |
| --- | --- | --- | --- | --- | --- | --- | --- | --- |
|  |  |  | **PubMed/Medline** | **Embase** | **CENTRAL, The Cochrane Library** | **LILACS** | **Cochrane infectious diseases group's trials register** | **Others** |
| Annane 2004(Annane et al, 2004) | - | - | + | + | + | + | + | - |
| Burry 2004(Burry & Wax, 2004) | NR | NR | + | + | + | - | - | - |
| Minneci 2004(Minneci et al, 2004) | NR | + | + | - | - | - | - | - |
| Annane 2009(Annane et al, 2009) | - | - | + | + | + | + | + | - |
| Sligl 2009(Sligl et al, 2009) | + | + | + | + | + | - | - | +^a^ |
| Moran 2010(Moran et al, 2010) | + | + | + | + | + | - | - | +^b^ |
| Wang 2014(Wang et al, 2014) | - | NR | + | + | + | + | + | +^c^ |
| Volbeda 2015(Volbeda et al, 2015) | - | - | + | + | + | - | - | +^d^ |
| Annane 2015(Annane et al, 2015) | - | - | + | + | + | + | + | - |
| Rochwerg 2018(Rochwerg et al, 2018) | - | - | + | + | + | + | - | - |
| Rygard 2018(Rygard et al, 2018) | - | NR | + | + | + | - | - | - |
| Xu 2018(Xu et al, 2018) | NR | NR | + | + | + | - | - | +^e^ |
| Zhu 2018(Zhu et al, 2018) | - | + | + | + | + | - | - | +^f^ |
| Fang 2019(Fang et al, 2019) | - | - | + | + | + | - | - | - |
| Lyu 2018(Lyu et al, 2018) | +^*^ | NR | + | + | + | - | - | +^g^ |
| Ni 2018(Ni et al, 2018) | - | - | + | + | + | - | - | +^h^ |

**NOTE:** NR: Not reported. CENTRAL: Cochrane Central Register of Controlled Trials. LILACS: Latin American Caribbean Health Sciences Literature. Others indicate that included study also used other search database except database listed in this Table. ^a^ Google scholar for clinical trials, Web of Science. ^b^ EBSCO Cinahl, America College of Physicians Journal Club, Health Technology Assessment Database, Database of Abstracts of Reviews of Effects. ^c^ Web of Knowledge. ^d^ Web of Science, Cinahl, clinicaltrials. gov. ^e^ Chinese Biological Medical Database. ^f^ Web of Science. ^g^ Web of Science & ClinicalTrials.gov databases. ^h^ Web of Science & Information Sciences Institute. ^*^ Language was restricted to English and Chinese, others (+) with limitation in English only.

**Supplementary Table 2** Primary studies included in meta-analysis

| **Primary studies** | **Annane 2004(Annane *et al*, 2004)** | **Burry 2004(Burry & Wax, 2004)** | **Minneci 2004(Minneci *et al*, 2004)** | **Annane 2009(Annane *et al*, 2009)** | **Sligl 2009(Sligl *et al*, 2009)** | **Moran 2010(Moran *et al*, 2010)** | **Wang 2014(Wang *et al*, 2014)** | **Volbeda 2015(Volbeda *et al*, 2015)** | **Annane 2015(Annane *et al*, 2015)** | **Rochwerg 2018(Rochwerg *et al*, 2018)** | **Rygard 2018(Rygard *et al*, 2018)** | **Xu 2018(Xu *et al*, 2018)** | **Zhu 2018(Zhu *et al*, 2018)** | **Fang 2019 (Fang *et al, 2019*)** | **Lyu**  **2018**  **(Lyu *et al, 2018*)** | **Ni**  **2018 (Ni *et al, 2018*)** |
| --- | --- | --- | --- | --- | --- | --- | --- | --- | --- | --- | --- | --- | --- | --- | --- | --- |
| Wagner 1955 | + | - | - | + | - | - | - | - | - | - | - | - | - | - | - | - |
| Bennett 1963 | - | - | + | - | - | - | - | - | - | - | - | - | - | - | - | - |
| CSG 1963 | + | - | - | - | - | + | - | - | + | + | + | - | - | - | - | - |
| Klastersky 1971 | + | - | + | + | - | + | - | + | - | - | - | - | - | + | - | - |
| Schumer 1976 | + | - | + | + | - | + | - | + | + | + | - | - | + | + | - | - |
| Thompson 1976 | - | - | + | - | - | - | - | - | - | - | - | - | - | - | - | - |
| Sprung 1984 | + | - | + | + | - | + | - | + | + | + | - | - | + | + | - | - |
| Lucas 1984 | + | - | + | + | - | - | - | - | - | - | - | - | - | - | - | -- |
| Hughes 1984 | - | - | - | - | - | - | - | + | - | - | - | - | - | - | - | - |
| Hoffman 1984 | - | - | - | - | - | - | - | + | - | - | - | - | - | - | - | - |
| Bone 1987 | + | - | + | + | - | + | - | + | + | + | - | - | + | + | - | - |
| VASSCSG 1987 | + | - | + | + | - | - | - | + | + | + | - | - | - | + | - | - |
| Bernard 1987 | - | - | - | - | - | - | - | + | - | - | - | - | - | - | - | - |
| Luce 1988 | + | - | + | + | - | + | - | + | + | + | - | - | + | + |  | - |
| Marik 1993 | - | - | - | - | - | - | - | + | - | - | - | - | - | - |  | - |
| Slusher 1996 | + | - | - | - | - | - | - | - | + | + | - | - | - | - |  | -- |
| Bollaert 1998 | + | + | + | + | + | + | + | + | + | + | + | + | + | + | + | + |
| Briegel 1999 | + | + | + | + | + | + | + | + | + | + | + | + | + | + | + | + |
| Chawla 1999 | + | + | + | + | + | + | + | + | + | + | + | - | + | + | - | - |
| Keh 1999 | - | + | - | - | - | - | - | - | - | - | - | - | - | - | - | - |
| Annane 2002 | + | + | + | + | + | + | + | + | + | + | + | + | + | + | + | + |
| Yildiz 2002 | + | + | + | + | - | + | - | + | + | + | - | - | - | + | - | + |
| de Gans 2002 | - | - | - | - | - | - | - | + | - | - | - | - | - | - | - | - |
| Keh 2003 | + | - | - | + | - | - | - | - | + | - | - | - | - | + | - | - |
| Thwaites 2004 | - | - | - | - | - | - | - | + | - | - | - | - | - | - | - | - |
| Oppert 2005 | - | - | - | + | + | + | + | + | + | + | + | + | - | + | + | + |
| Tandan 2005 | - | - | - | + | - | + | - | - | + | + | + | - | - | + | - | - |
| Levy 2005 | - | - | - | - | + | - | - | - | - | - | - | - | - | - | - | - |
| Mussack 2005 | - | - | - | - | - | - | + | - | - | - | - | - | - | - | - | - |
| Huh 2006 | - | - | - | + | - | - | - | - | + | - | - | - | - | - | - | - |
| Rinaldi 2006 | - | - | - | + | - | - | - | + | + | + | - | - | - | + | - | + |
| Cicarelli 2006 | - | - | - | - | - | - | - | + | - | - | - | - | - | - | - | - |
| Cicarelli 2007 | - | - | - | + | - | - | - | + | + | + | + | + | + | + | - | + |
| Meduri 2007 | - | - | - | + | - | - | - | - | + | + | - | - | - | + | - | + |
| Raurich 2007 | - | - | - | - | + | - | - | - | - | - | - | - | - | - | - | - |
| Scarborough 2007 | - | - | - | - | - | - | - | + | - | - | - | - | - | - | - | - |
| Sprung 2008 | - | - | - | + | + | + | + | + | + | + | + | + | + | + | + | + |
| Confalonieri 2005 | - | - | - | + | - | - | - | + | + | + | - | - | - | + | - | + |
| Kaufmann 2008 | - | - | - | - | - | - | - | + | - | - | - | - | - | - | - | - |
| Aboab 2008 | - | - | - | - | - | - | - | - | - | - | + | - | - | + | - | - |
| Kurugundla 2008 | - | - | - | - | - | - | - | - | - | - | + | - | - | - | - | - |
| Hu 2009 | - | - | - | - | - | - | - | + | + | + | - | - | + | + | + | + |
| Meduri 2009 | - | - | - | - | - | - | - | + | - | + | + | - | - | - | - | - |
| Valoor 2009 | - | - | - | - | - | - | - | - | + | + | - | - | - | - | - | - |
| Arabi 2010 | - | - | - | - | - | - | + | + | + | + | + | + | + | + | + | + |
| Snijders 2010 | - | - | - | - | - | - | - | + | + | + | - | - | - | + | - | - |
| Wan 2011 | - | - | - | - | - | - | - | + | - | - | - | + | - | - | - | - |
| Yildiz 2011 | - | - | - | - | - | - | - | + | + | + | - | - | - | + | - | + |
| Meijvis 2011 | - | - | - | - | - | - | - | - | + | + | - | - | - | + | - | - |
| Sabry 2011 | - | - | - | - | - | - | - | - | + | + | - | - | - | + | - | -- |
| Liu 2012 | - | - | - | - | - | - | - | - | + | + | - | - | - | + | - | - |
| Nafae 2013 | - | - | - | - | - | - | - | + | - | - | - | - | - | - | - | - |
| Rezk 2013 | - | - | - | - | - | - | - | - | + | + | - | - | - | + | - | - |
| Gordon 2014 | - | - | - | - | - | - | - | + | + | + | + | + | + | + | + | + |
| Mirea 2014 | - | - | - | - | - | - | - | + | - | + | + | - | - | - | - | - |
| Ruolan 2014 | - | - | - | - | - | - | - | + | - | - | - | - | - | - | - | - |
| Branco 2014 | - | - | - | - | - | - | - | - | - | + | - | - | - | - | - | - |
| De Graaf 2014 | - | - | - | - | - | - | - | - | - | + | - | - | - | - | - | - |
| Huang 2014 | - | - | - | - | - | - | - | - | - | - | - | - | - | + | - | - |
| Torres 2015 | - | - | - | - | - | - | - | - | + | + | + | - | - | + | - | - |
| Annane 2015 | - | - | - | - | - | - | - | - | - | - | + | - | - | - | - | - |
| Povoa 2015 | - | - | - | - | - | - | - | - | - | - | - | - | + | - | - | - |
| Keh 2016 | - | - | - | - | - | - | - | - | - | + | - | - | + | + | - | + |
| Gorden 2016 | - | - | - | - | - | - | - | - | - | + | + | - | - | + | + | - |
| Tongyoo 2016 | - | - | - | - | - | - | - | - | - | + | + | - | - | + | + | + |
| EI-Nawawy 2017 | - | - | - | - | - | - | - | - | - | + | - | - | - | - | - | - |
| Lv 2017 | - | - | - | - | - | - | - | - | - | + | + | - | + | + | + | + |
| Menon 2017 | - | - | - | - | - | - | - | - | - | + | - | - | - | - | - | - |
| Venkatesh 2018 | - | - | - | - | - | - | - | - | - | + | + | - | + | + | + | + |
| Annane 2018 | - | - | - | - | - | - | - | - | - | + | + | - | + | + | + | + |

**Note:** V.A.: The Veterans Administration Systemic Sepsis Cooperative Study Group. CSG: Cooperative Study Group. VASSCSG: Veterans Administration Systemic Sepsis Cooperative Study Group.

**Supplementary Table 3** Outcomes reported by individual included study

| **Meta-analysis** | **28-day mortality** | **90-day mortality** | **ICU mortality** | **Hospital mortality** | **Shock reversal at day 7** | **Shock reversal at 28 days** | **Length of stay in ICU** | **Length of stay in hospital** | **Number of organs affected and severity of organ** | **Others outcomes** |
| --- | --- | --- | --- | --- | --- | --- | --- | --- | --- | --- |
| Annane 2004(Annane *et al*, 2004) | + | - | + | + | + | + | - | - | - | - |
| Burry 2004(Burry & Wax, 2004) | + | - | - | - | - | - | - | - | - | - |
| Minneci* 2004(Minneci *et al*, 2004) | + | - | - | - | +^a^ | - | - | - | - | - |
| Annane 2009(Annane *et al*, 2009) | + | - | + | + | + | + | + | - | - | - |
| Sligl 2009(Sligl *et al*, 2009) | + | - | - | - | + | - | - | - | - | - |
| Moran^#^2010(Moran *et al*, 2010) | - | - | - | - | +^a^ | - | - | - | - | + |
| Wang 2014(Wang *et al*, 2014) | + | - | - | - | + | + | - | - | - | - |
| Volbeda2015(Volbeda *et al*, 2015) | +^b^ | + | - | - | - | - | - | - | - | + |
| Annane 2015(Annane *et al*, 2015) | + | - | + | + | + | + | + | + | + | + |
| Rochwerg2018(Rochwerg *et al*, 2018) | +^c^ | - | - | - | + | - | + | + | - | + |
| Rygard 2018(Rygard *et al*, 2018) | - | - | - | - | - | - | + | + | - | + |
| Xu 2018(Xu *et al*, 2018) | + | - | - | - | + | - | - | - | - | - |
| Zhu 2018(Zhu *et al*, 2018) | + | + | + | + | +^a^ | - | + | + | - | - |
| Fang 2019(Fang et al, 2019) | + | + | + | + | + | - | + | + | + | + |
| Lyu 2018(Lyu et al, 2018) | + | - | + | + | + ^a^ | - | + | - | - | + |
| Ni 2018(Ni et al, 2018) | + | - | - | - | - | - | - | - | - | + |

**Note:** ICU: intensive care unit. * indicates that use effective size of the Relative Survival Benefit. # indicates that use effective size of the odds ratio.  ^a^ only reported the shock reversal and didn't declaim the time of shock reversal. ^b^ reported mortality of 30 days but was regarded as mortality in 28 days. ^c^ reported mortality from 28 to 30 days and was regard as mortality in 28 days.

**Supplementary Table 4**Adverse events included in meta-analysis

| **Meta-analysis** | **Gastrointestinal bleeding** | **Superinfection** | **Hyperglycaemia** | **Hypernatraemia** | **Neuromuscular weakness** | **ICU acquired bactriemia** | **Neuropsychiatric outcomes** |
| --- | --- | --- | --- | --- | --- | --- | --- |
| Annane2004(Annane *et al*, 2004) | + | + | + | + | - | - | - |
| Burry 2004(Burry & Wax, 2004) | + | + | - | - | - | - | - |
| Minneci2004(Minneci *et al*, 2004) | - | + | - | - | - | - | - |
| Annane 2009(Annane *et al*, 2009) | + | + | + | + | + | - | - |
| Sligl 2009(Sligl *et al*, 2009) | - | + | - | - | - | - | - |
| Moran 2010(Moran *et al*, 2010) | + | + | + | - | - | - | - |
| Wang 2014(Wang *et al*, 2014) | + | + | + | - | - | - | - |
| Volbeda *2015(Volbeda *et al*, 2015) | - | - | - | - | - | + | - |
| Annane 2015(Annane *et al*, 2015) | + | + | + | + | + | - | - |
| Rochwerg 2018(Rochwerg *et al*, 2018) | + | + | + | + | - | - | + |
| Rygard 2018(Rygard *et al*, 2018) | + | + | + | + | - | - | +^#^ |
| Xu 2018(Xu *et al*, 2018) | + | + | - | - | - | - | - |
| Zhu2018(Zhu *et al*, 2018) | - | + | - | - | - | - | - |
| Fang 2019(Fang et al, 2019) | + | + | + | + | - | - | - |
| Lyu 2018(Lyu et al, 2018) | + | + | + | - | - | - | - |
| Ni 2018(Ni et al, 2018) | - | - | - | - | - | - | - |

**Note:** ICU: intensive care unit. * indicates that only reported total incidence of adverse events. ^#^ indicates that incidence of delirium or encephalopathy.

**Supplementary Table 5** Heterogeneity or subgroup analyses of primary studies

| **Items of subgroup or sensitivity analysis** | **Annane 2004(Annane *et al*, 2004)** | | **Burry 2004(Burry & Wax, 2004)** | **Minneci 2004(Minneci *et al*, 2004)** | **Annane 2009(Annane *et al*, 2009)** | **Sligl 2009(Sligl *et al*, 2009)** | **Moran 2010(Moran *et al*, 2010)** | **Wang 2014(Wang *et al*, 2014)** | **Volbeda 2015(Volbeda *et al*, 2015)** | **Annane 2015(Annane *et al*, 2015)** | **Rochwerg 2018(Rochwerg *et al*, 2018)** | **Rygard 2018(Rygard *et al*, 2018)** | **Xu**  **2018(Xu *et al*, 2018)** | **Zhu 2018(Zhu *et al*, 2018)** | **Fang 2019(Fang et al, 2019)** | **Lyu 2018(Lyu et al, 2018)** | **Ni 2018(Ni et al, 2018)** |
| --- | --- | --- | --- | --- | --- | --- | --- | --- | --- | --- | --- | --- | --- | --- | --- | --- | --- |
| Statistical heterogeneity analysis | + | | 0 | + | + | + | + | + | + | + | + | + | + | + | + | + | + |
|  | |  |  |  |  |  |  |  |  |  |  |  |  |  |  |  |  |
| Primary study quality | + | | + | + | + | + | + | + | + | + | + | + | + | + | + | + | + |
| Size of primary study | - | | 0 | + | - | - | + | + | 0 | 0 | - | - | - | + | + | + | + |
| Publication bias of primary study | + | | - | - | + | + | + | + | + | + | + | + | + | + | + | + | + |
| The duration or/and dose of corticosteroids | + | | + | + | + | + | + | + | + | + | + | + | + | + | + | - | - |
| Mortality on different day | + | | + | - | + | + | - | + | + | + | + | + | + | + | + | - | - |
| Mortality of ICU on different dose | - | | - | - | + | - | - | - | - | - | - | - | - | - | - | - | - |
| Mortality of Hospital on different dose | + | | - | - | + | - | - | - | - | + | - | - | - | + | - | - |  |
| The length of ICU and Hospital on different dose | - | | - | - | - | - | - | - | - | - | - | - | - | + | - | - | - |
| Shock reversal at different day | + | | - | - | + | + | - | + | - | + | + | - | + | - | - | - | - |
| Adverse events | + | | + | + | + | + | + | + | + | + | + | + | + | + | + | + | + |

**Note:** “+/-” indicates that formal sensitivity or subgroup analyses were not performed; “0” indicates that descriptive data were performed or discussed, but not analysis was performed.

**References**

Annane D, Bellissant E, Bollaert PE, Briegel J, Confalonieri M, De Gaudio R, et al. (2009). Corticosteroids in the treatment of severe sepsis and septic shock in adults: a systematic review. Jama.301: 2362-75.

Annane D, Bellissant E, Bollaert PE, Briegel J, Keh D, Kupfer Y (2004). Corticosteroids for severe sepsis and septic shock: a systematic review and meta-analysis. Bmj.329: 480.

Annane D, Bellissant E, Bollaert PE, Briegel J, Keh D, Kupfer Y (2015). Corticosteroids for treating sepsis. Cochrane Database Syst Rev: Cd002243.

Burry LD, Wax RS (2004). Role of corticosteroids in septic shock. Ann Pharmacother.38: 464-72.

Lin L-L, Gu H-Y, Luo J, Wu J-Y , Wang L, Zuo H-X, et al. (2018). Impact of corticosteroids on short- and long-term mortality in patients with sepsis and septic shock: a meta-analysis with trial sequential analysis. Under Review

Minneci PC, Deans KJ, Banks SM, Eichacker PQ, Natanson C (2004). Meta-analysis: the effect of steroids on survival and shock during sepsis depends on the dose. Ann Intern Med.141: 47-56.

Moran JL, Graham PL, Rockliff S, Bersten AD (2010). Updating the evidence for the role of corticosteroids in severe sepsis and septic shock: a Bayesian meta-analytic perspective. Crit Care.14: R134.

Rochwerg B, Oczkowski SJ, Siemieniuk RAC, Agoritsas T, Belley-Cote E, D'Aragon F, et al. (2018). Corticosteroids in Sepsis: An Updated Systematic Review and Meta-Analysis. Crit Care Med

Rygard SL, Butler E, Granholm A, Moller MH, Cohen J, Finfer S (2018). Low-dose corticosteroids for adult patients with septic shock: a systematic review with meta-analysis and trial sequential analysis.

Sligl WI, Milner DA, Jr., Sundar S, Mphatswe W, Majumdar SR (2009). Safety and efficacy of corticosteroids for the treatment of septic shock: A systematic review and meta-analysis. Clin Infect Dis.49: 93-101.

Volbeda M, Wetterslev J, Gluud C, Zijlstra JG, van der Horst IC, Keus F (2015). Glucocorticosteroids for sepsis: systematic review with meta-analysis and trial sequential analysis. Intensive Care Med.41: 1220-34.

Wang C, Sun J, Zheng J, Guo L, Ma H, Zhang Y, et al. (2014). Low-dose hydrocortisone therapy attenuates septic shock in adult patients but does not reduce 28-day mortality: a meta-analysis of randomized controlled trials. Anesth Analg.118: 346-57.

Xu R, Wang Q, Huang Y, Wu L, Liu Q, Hu W, et al. (2018). Do low-dose corticosteroids improve survival or shock reversal from septic shock in adults? Meta-analysis with trial sequential analysis. J Int Med Res.46: 2513-24.

Zhu Y, Wen Y, Jiang Q, Guo N, Cai Y, Shen X (2018). The Effectiveness and Safety of Corticosteroids Therapy in Adult Critical Ill Patients with Septic Shock: A Meta-Analysis of Randomized Controlled Trials. Shock

Fang F, Zhang Y, Tang J-J, Lunsford L-D, Li T-G, et al. (2019). Association of Corticosteroid Treatment With Outcomes in Adult Patients With Sepsis: A Systematic Review and Meta-analysis. JAMA Intern Med. 179(2):213-223.

Lyu Q-Q, Chen Q-H, Zheng R-Q, Yu J-Q, Gu X-H. (2018). Effect of Low-Dose Hydrocortisone Therapy in Adult Patients With Septic Shock: A Meta-Analysis With Trial Sequential Analysis of Randomized Controlled Trials. J of Intensive Care Med.

Ni Y-N, Liu Y- M, Wang Y-W, Liang B-M, Liang Z-A. (2018).Can corticosteroids reduce the mortality of patients with severe sepsis? A systematic review and meta-analysis. Am J Emerg Med. 27.
